# (Pyridin-4-yl)methyl *N*′-(3-phenyl­allyl­idene)hydrazinecarbodithio­ate

**DOI:** 10.1107/S1600536812051537

**Published:** 2013-01-04

**Authors:** May Lee Low, Thahira Begum S. A. Ravoof, Mohamed Ibrahim Mohamed Tahir, Karen A. Crouse, Edward R. T. Tiekink

**Affiliations:** a Department of Chemistry, Universiti Putra Malaysia, 43400 Serdang, Malaysia; b Department of Chemistry, University of Malaya, 50603 Kuala Lumpur, Malaysia

## Abstract

In the title compound, C_16_H_15_N_3_S_2_, the central C_2_N_2_S_2_ residue is planar (r.m.s. deviation = 0.045 Å) and the pyridyl and benzene rings are inclined and approximately coplanar to this plane, respectively [dihedral angles = 72.85 (9) and 10.73 (9)°], so that, overall, the mol­ecule adopts an L-shape. The conformation about each of the N=C [1.290 (3) Å] and C=C [1.340 (3) Å] bonds is *E*. Supra­molecular chains along [1-10] are stabilized by N—H⋯N(pyridine) hydrogen bonding and these are connected into a double layer that stacks along the *c-*axis direction by C—H⋯π(pyridine) inter­actions.

## Related literature

For background to related Schiff bases of S-substituted dithio­carbaza­tes with cinnamaldehyde, see: Tarafder *et al.* (2008 ▶, 2010 ▶). For the corresponding metal complexes, see: Reza *et al.* (2012 ▶); Liu *et al.* (2009 ▶). For the biological activity of similar sulfur–nitro­gen-containing Schiff base derivatives, see: Maia *et al.* (2010 ▶); Pavan *et al.* (2010 ▶); Zhu *et al.* (2009 ▶). For the synthesis, see: Crouse *et al.* (2004 ▶); Khoo (2008 ▶); Tarafder *et al.* (2008 ▶, 2010 ▶).
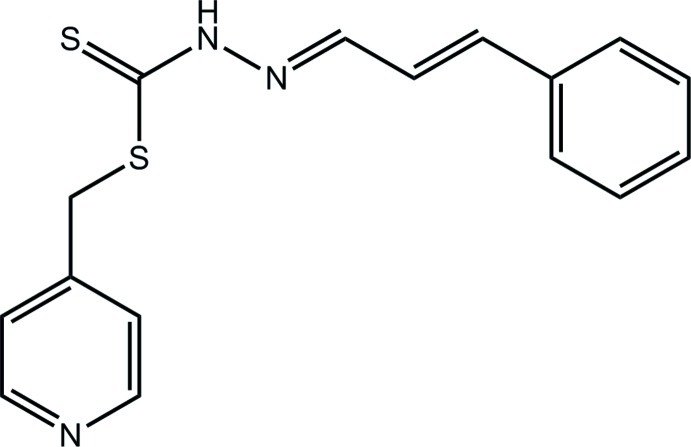


## Experimental

### 

#### Crystal data

C_16_H_15_N_3_S_2_
*M* *_r_* = 313.43
Triclinic, 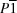

*a* = 5.3784 (5) Å
*b* = 10.1570 (9) Å
*c* = 14.5488 (17) Å
α = 77.315 (9)°
β = 84.735 (9)°
γ = 78.193 (8)°
*V* = 758.06 (13) Å^3^
*Z* = 2
Cu *K*α radiation
μ = 3.14 mm^−1^
*T* = 100 K
0.13 × 0.06 × 0.01 mm

#### Data collection

Oxford Diffraction Xcaliber Eos Gemini diffractometer
Absorption correction: multi-scan (*CrysAlis PRO*; Agilent, 2011 ▶) *T*_min_ = 0.83, *T*_max_ = 0.97
15664 measured reflections
2918 independent reflections
2469 reflections with *I* > 2σ(*I*)
*R*_int_ = 0.045

#### Refinement

*R*[*F* ^2^ > 2σ(*F* ^2^)] = 0.042
*wR*(*F* ^2^) = 0.115
*S* = 1.05
2918 reflections
193 parameters
1 restraint
H atoms treated by a mixture of independent and constrained refinement
Δρ_max_ = 0.42 e Å^−3^
Δρ_min_ = −0.29 e Å^−3^

### 

Data collection: *CrysAlis PRO* (Agilent, 2011 ▶); cell refinement: *CrysAlis PRO*; data reduction: *CrysAlis PRO*; program(s) used to solve structure: *SHELXS97* (Sheldrick, 2008 ▶); program(s) used to refine structure: *SHELXL97* (Sheldrick, 2008 ▶); molecular graphics: *ORTEP-3 for Windows* (Farrugia, 2012 ▶) and *DIAMOND* (Brandenburg, 2006 ▶); software used to prepare material for publication: *publCIF* (Westrip, 2010 ▶).

## Supplementary Material

Crystal structure: contains datablock(s) global, I. DOI: 10.1107/S1600536812051537/qm2090sup1.cif

Structure factors: contains datablock(s) I. DOI: 10.1107/S1600536812051537/qm2090Isup2.hkl

Supplementary material file. DOI: 10.1107/S1600536812051537/qm2090Isup3.cml

Additional supplementary materials:  crystallographic information; 3D view; checkCIF report

## Figures and Tables

**Table 1:** Hydrogen-bond geometry (Å, °) *Cg*1 is the centroid of the N3,C12–C15 pyridyl ring.

*D*—H⋯*A*	*D*—H	H⋯*A*	*D*⋯*A*	*D*—H⋯*A*
N1—H1*n*⋯N3^i^	0.88 (2)	2.02 (2)	2.897 (3)	172 (2)
C8—H8⋯*Cg*1^ii^	0.95	2.92	3.701 (3)	141

Symmetry codes: (i) 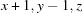; (ii) 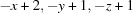.

**Fig. 1.**
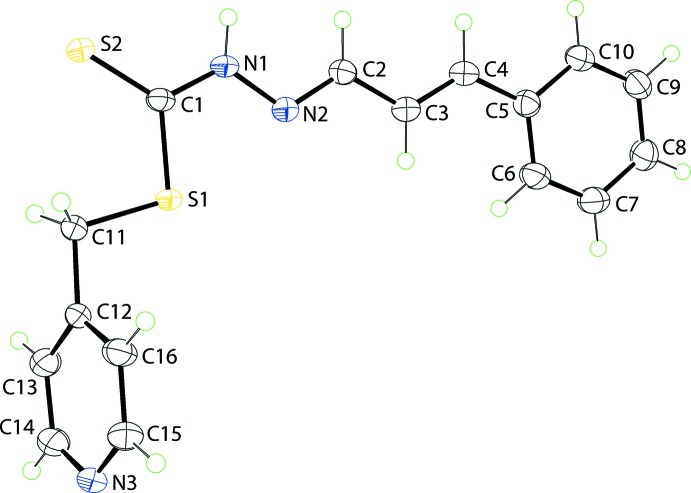
The molecular structure of (I) showing the atom-labelling scheme and displacement ellipsoids at the 50% probability level.

**Fig. 2.**
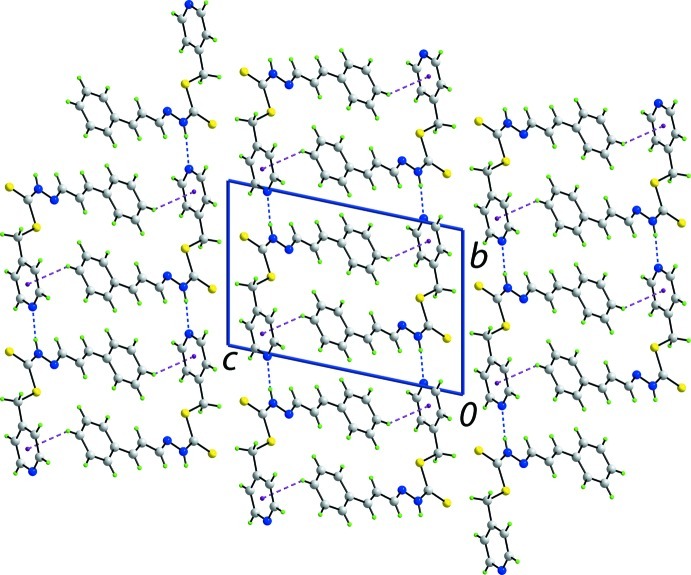
A view of the crystal packing in projection down the a axis, highlighting the stacking of supramolecular layers along the c axis. The N—H···N and C—H···π interactions are shown as blue and purple dashed lines, respectively.

## supplementary crystallographic information

### Comment

Schiff bases of *S*-substituted dithiocarbazates with cinnamaldehyde attract interest in terms of both coordination chemistry (Reza *et al.*, 2012; Liu *et al.*, 2009) and for their biological activities (Maia *et al.*, 2010; Pavan *et al.*, 2010; Zhu *et al.*, 2009. In pursuing our continuing interest in the coordination chemistry of dithiocarbazate derivatives and their biological importance (Tarafder *et al.*, 2010; Tarafder *et al.*, 2008), the title compound, (I), the product of condensation between *S*-4-picolyl dithiocarbazate and cinnamaldehyde, was investigated.

In (I), Fig. 1, the central C_2_N_2_S_2_ residue is planar (r.m.s. deviation = 0.045 Å) with maximum deviations of 0.040 (2) Å for each of N1 and C11, and -0.048 (1) Å for the S1 atom. The pyridyl ring is inclined to this plane, forming a dihedral angle of 72.85 (9) °, whereas the benzene ring is almost co-planar [dihedral angle = 10.73 (9)°]. The maximum twist from co-planarity along the C_5_N_2_ chain is seen in the C1—N1—N2—C2 torsion angle of -176.15 (19) Å. The amine-N1—H atom is *syn* to the thione-S2 atom. The conformation about each of the N2═C2 [1.290 (3) Å] and C3═C4 [1.340 (3) Å] bonds is *E*. Globally, the molecule adopts an *L*-shape as the pyridyl residue is *anti* to the thione-S2 atom. A very similar conformation was found in the benzyl ester (Tarafder *et al.*, 2008).

The pyridyl ring proves pivotal in the crystal packing by forming a hydrogen bond with the amine-N1—H atom and acting as an acceptor in a C—H···π(pyridyl) contact, Table 1. The hydrogen bonding leads to the formation of supramolecular chains along [1 1 0] and these are connected into a double layer in the *ab* plane *via* the C—H···π(pyridyl) contacts, Fig. 2.

### Experimental

The previously reported method for preparation substituted dithiocarbazate (Crouse *et al.*, 2004) was modified by reaction with 4-picolylchloride hydrochloride (Khoo, 2008).

Potassium hydroxide (11.4 g, 0.2 mol) was dissolved completely in 90% ethanol (70 ml) and the mixture was cooled in ice. To the cold solution, hydrazine hydrate (9.7 ml, 0.2 mol) was added slowly with stirring. Carbon disulfide (12.0 ml, 0.2 mol) was then added drop-wise with vigorous stirring for about 1 h. The temperature of the reaction mixture was kept below 268 K during addition. During this time two layers formed. The resulting yellow oil (lower layer) was separated and dissolved in 40% ethanol (60 ml). 4-Picolylchloride hydrochloride (32.8 g, 0.2 mol) was completely dissolved in 100 ml of 80% ethanol and added slowly to the above solution with vigorous mechanical stirring. The resulting white product (S4PDTC) was separated by filtration, washed with water and dried. The crude product was recrystallized from absolute ethanol.

Previously reported methods for preparation of Schiff bases (Tarafder *et al.*, 2010; Tarafder *et al.*, 2008) were used to prepare S4PDTC derivatives with cinnamaldehyde. An equimolar amount of cinnamaldehyde (1.26 ml) was added to the solution of S4PDTC (1.99 g, 0.01 mol) dissolved in hot absolute ethanol (100 ml). The mixture was heated while being stirred to reduce it to half the original volume and then cooled. The orange compound was filtered, washed with absolute ethanol then dried over silica gel. Single crystals were obtained after recrystallization from a mixture of DMF/chloroform. (yield 72%, *M*.pt: 481–482 K).

### Refinement

Carbon-bound H-atoms were placed in calculated positions (C—H 0.95 to 0.99 Å) and were included in the refinement in the riding model approximation, with *U*_iso_(H) = 1.2*U*_equiv_(C). The nitrogen-bound H-atom was refined with N—H = 0.88±0.01 Å. The (0 3 14) reflection was omitted from the final refinement owing to poor agreement.

### Crystal data

**Table:**

C_16_H_15_N_3_S_2_	*Z* = 2
*M**_r_* = 313.43	*F*(000) = 328
Triclinic, *P*1	*D*_x_ = 1.373 Mg m^−^^3^
Hall symbol: -P 1	Cu *K*α radiation, λ = 1.54184 Å
*a* = 5.3784 (5) Å	Cell parameters from 5833 reflections
*b* = 10.1570 (9) Å	θ = 3–72°
*c* = 14.5488 (17) Å	µ = 3.14 mm^−^^1^
α = 77.315 (9)°	*T* = 100 K
β = 84.735 (9)°	Thin-plate, orange
γ = 78.193 (8)°	0.13 × 0.06 × 0.01 mm
*V* = 758.06 (13) Å^3^	

### Data collection

**Table:**

Oxford Diffraction Xcaliber Eos Gemini diffractometer	2918 independent reflections
Radiation source: fine-focus sealed tube	2469 reflections with *I* > 2σ(*I*)
Graphite monochromator	*R*_int_ = 0.045
Detector resolution: 16.1952 pixels mm^-1^	θ_max_ = 72.3°, θ_min_ = 3.1°
ω scans	*h* = −6→6
Absorption correction: multi-scan (*CrysAlis PRO*; Agilent, 2011)	*k* = −12→12
*T*_min_ = 0.83, *T*_max_ = 0.97	*l* = −17→15
15664 measured reflections	

### Refinement

**Table:**

Refinement on *F*^2^	Primary atom site location: structure-invariant direct methods
Least-squares matrix: full	Secondary atom site location: difference Fourier map
*R*[*F*^2^ > 2σ(*F*^2^)] = 0.042	Hydrogen site location: inferred from neighbouring sites
*wR*(*F*^2^) = 0.115	H atoms treated by a mixture of independent and constrained refinement
*S* = 1.05	*w* = 1/[σ^2^(*F*_o_^2^) + (0.0669*P*)^2^ + 0.3038*P*] where *P* = (*F*_o_^2^ + 2*F*_c_^2^)/3
2918 reflections	(Δ/σ)_max_ < 0.001
193 parameters	Δρ_max_ = 0.42 e Å^−^^3^
1 restraint	Δρ_min_ = −0.29 e Å^−^^3^

### Special details

**Table:**

Geometry. All s.u.'s (except the s.u. in the dihedral angle between two l.s. planes) are estimated using the full covariance matrix. The cell s.u.'s are taken into account individually in the estimation of s.u.'s in distances, angles and torsion angles; correlations between s.u.'s in cell parameters are only used when they are defined by crystal symmetry. An approximate (isotropic) treatment of cell s.u.'s is used for estimating s.u.'s involving l.s. planes.
Refinement. Refinement of *F*^2^ against ALL reflections. The weighted *R*-factor *wR* and goodness of fit *S* are based on *F*^2^, conventional *R*-factors *R* are based on *F*, with *F* set to zero for negative *F*^2^. The threshold expression of *F*^2^ > 2σ(*F*^2^) is used only for calculating *R*-factors(gt) *etc*. and is not relevant to the choice of reflections for refinement. *R*-factors based on *F*^2^ are statistically about twice as large as those based on *F*, and *R*- factors based on ALL data will be even larger.

### Fractional atomic coordinates and isotropic or equivalent isotropic displacement parameters (Å^2^)

**Table:**

	*x*	*y*	*z*	*U*_iso_*/*U*_eq_	
S1	0.36876 (9)	0.53086 (5)	0.18295 (4)	0.02532 (16)	
S2	0.33320 (10)	0.32045 (5)	0.06781 (4)	0.02701 (17)	
N1	0.6775 (3)	0.29601 (17)	0.19095 (13)	0.0256 (4)	
H1n	0.749 (4)	0.2148 (14)	0.1794 (18)	0.031*	
N2	0.7786 (3)	0.34184 (17)	0.25917 (13)	0.0262 (4)	
N3	−0.1177 (4)	1.01818 (18)	0.16683 (15)	0.0338 (5)	
C1	0.4717 (4)	0.3730 (2)	0.14713 (15)	0.0233 (4)	
C2	0.9846 (4)	0.2638 (2)	0.29282 (16)	0.0266 (5)	
H2	1.0579	0.1846	0.2682	0.032*	
C3	1.1038 (4)	0.2962 (2)	0.36748 (16)	0.0284 (5)	
H3	1.0295	0.3764	0.3909	0.034*	
C4	1.3162 (4)	0.2171 (2)	0.40512 (16)	0.0279 (5)	
H4	1.3893	0.1405	0.3776	0.033*	
C5	1.4484 (4)	0.2350 (2)	0.48372 (16)	0.0284 (5)	
C6	1.3649 (5)	0.3430 (2)	0.53137 (18)	0.0370 (6)	
H6	1.2139	0.4074	0.5133	0.044*	
C7	1.4982 (5)	0.3574 (3)	0.60420 (19)	0.0393 (6)	
H7	1.4401	0.4323	0.6349	0.047*	
C8	1.7160 (5)	0.2633 (3)	0.63253 (18)	0.0368 (6)	
H8	1.8074	0.2733	0.6826	0.044*	
C9	1.8000 (4)	0.1543 (3)	0.58746 (18)	0.0359 (5)	
H9	1.9484	0.0889	0.6072	0.043*	
C10	1.6683 (4)	0.1405 (2)	0.51347 (17)	0.0312 (5)	
H10	1.7283	0.0659	0.4827	0.037*	
C11	0.1118 (4)	0.6125 (2)	0.10459 (17)	0.0269 (5)	
H11A	0.1725	0.6164	0.0379	0.032*	
H11B	−0.0298	0.5612	0.1181	0.032*	
C12	0.0263 (4)	0.7559 (2)	0.12385 (16)	0.0262 (5)	
C13	−0.1950 (4)	0.7908 (2)	0.17696 (19)	0.0357 (6)	
H13	−0.3022	0.7258	0.1998	0.043*	
C14	−0.2588 (4)	0.9219 (2)	0.1965 (2)	0.0390 (6)	
H14	−0.4112	0.9441	0.2330	0.047*	
C15	0.0961 (5)	0.9839 (2)	0.11574 (18)	0.0345 (5)	
H15	0.1999	1.0508	0.0940	0.041*	
C16	0.1742 (4)	0.8555 (2)	0.09283 (17)	0.0314 (5)	
H16	0.3277	0.8361	0.0562	0.038*	

### Atomic displacement parameters (Å^2^)

**Table:**

	*U*^11^	*U*^22^	*U*^33^	*U*^12^	*U*^13^	*U*^23^
S1	0.0270 (3)	0.0170 (3)	0.0333 (3)	0.00106 (19)	−0.0078 (2)	−0.0104 (2)
S2	0.0278 (3)	0.0215 (3)	0.0342 (3)	−0.0011 (2)	−0.0060 (2)	−0.0124 (2)
N1	0.0275 (9)	0.0184 (8)	0.0315 (10)	0.0004 (7)	−0.0035 (8)	−0.0100 (7)
N2	0.0260 (9)	0.0226 (9)	0.0312 (10)	−0.0014 (7)	−0.0049 (7)	−0.0095 (7)
N3	0.0309 (10)	0.0233 (9)	0.0484 (13)	0.0048 (7)	−0.0124 (9)	−0.0149 (9)
C1	0.0249 (10)	0.0176 (9)	0.0272 (11)	−0.0021 (8)	0.0002 (8)	−0.0062 (8)
C2	0.0251 (10)	0.0224 (10)	0.0323 (12)	−0.0021 (8)	−0.0013 (9)	−0.0078 (9)
C3	0.0292 (11)	0.0234 (10)	0.0334 (12)	−0.0025 (8)	−0.0023 (9)	−0.0096 (9)
C4	0.0300 (11)	0.0238 (10)	0.0299 (12)	−0.0020 (8)	0.0003 (9)	−0.0089 (9)
C5	0.0271 (11)	0.0262 (11)	0.0313 (12)	−0.0045 (8)	−0.0021 (9)	−0.0051 (9)
C6	0.0370 (13)	0.0302 (12)	0.0429 (15)	0.0047 (9)	−0.0111 (11)	−0.0122 (10)
C7	0.0419 (14)	0.0341 (13)	0.0436 (15)	0.0009 (10)	−0.0083 (11)	−0.0166 (11)
C8	0.0345 (12)	0.0433 (14)	0.0341 (13)	−0.0045 (10)	−0.0093 (10)	−0.0111 (11)
C9	0.0281 (11)	0.0395 (13)	0.0387 (14)	0.0020 (9)	−0.0057 (10)	−0.0111 (11)
C10	0.0291 (11)	0.0303 (11)	0.0339 (13)	−0.0007 (9)	−0.0007 (9)	−0.0108 (9)
C11	0.0268 (10)	0.0201 (10)	0.0344 (12)	0.0004 (8)	−0.0097 (9)	−0.0077 (9)
C12	0.0270 (10)	0.0198 (10)	0.0320 (12)	0.0022 (8)	−0.0113 (9)	−0.0081 (8)
C13	0.0260 (11)	0.0314 (12)	0.0542 (16)	−0.0051 (9)	−0.0020 (10)	−0.0185 (11)
C14	0.0257 (11)	0.0359 (13)	0.0584 (17)	0.0018 (9)	−0.0017 (11)	−0.0235 (12)
C15	0.0389 (13)	0.0204 (10)	0.0439 (14)	−0.0034 (9)	−0.0068 (11)	−0.0060 (9)
C16	0.0328 (11)	0.0230 (10)	0.0363 (13)	0.0005 (9)	−0.0022 (10)	−0.0071 (9)

### Geometric parameters (Å, º)

**Table:**

S1—C1	1.760 (2)	C7—C8	1.383 (3)
S1—C11	1.817 (2)	C7—H7	0.9500
S2—C1	1.662 (2)	C8—C9	1.385 (4)
N1—C1	1.342 (3)	C8—H8	0.9500
N1—N2	1.379 (3)	C9—C10	1.389 (3)
N1—H1n	0.880 (10)	C9—H9	0.9500
N2—C2	1.290 (3)	C10—H10	0.9500
N3—C14	1.332 (3)	C11—C12	1.514 (3)
N3—C15	1.337 (3)	C11—H11A	0.9900
C2—C3	1.439 (3)	C11—H11B	0.9900
C2—H2	0.9500	C12—C13	1.385 (3)
C3—C4	1.340 (3)	C12—C16	1.386 (3)
C3—H3	0.9500	C13—C14	1.391 (3)
C4—C5	1.463 (3)	C13—H13	0.9500
C4—H4	0.9500	C14—H14	0.9500
C5—C10	1.398 (3)	C15—C16	1.389 (3)
C5—C6	1.400 (3)	C15—H15	0.9500
C6—C7	1.382 (3)	C16—H16	0.9500
C6—H6	0.9500		
			
C1—S1—C11	101.51 (10)	C9—C8—H8	120.2
C1—N1—N2	119.67 (17)	C8—C9—C10	120.2 (2)
C1—N1—H1n	122.4 (17)	C8—C9—H9	119.9
N2—N1—H1n	117.9 (17)	C10—C9—H9	119.9
C2—N2—N1	114.56 (18)	C9—C10—C5	120.9 (2)
C14—N3—C15	116.84 (19)	C9—C10—H10	119.6
N1—C1—S2	121.98 (16)	C5—C10—H10	119.6
N1—C1—S1	113.13 (16)	C12—C11—S1	105.44 (14)
S2—C1—S1	124.88 (12)	C12—C11—H11A	110.7
N2—C2—C3	120.4 (2)	S1—C11—H11A	110.7
N2—C2—H2	119.8	C12—C11—H11B	110.7
C3—C2—H2	119.8	S1—C11—H11B	110.7
C4—C3—C2	122.2 (2)	H11A—C11—H11B	108.8
C4—C3—H3	118.9	C13—C12—C16	117.4 (2)
C2—C3—H3	118.9	C13—C12—C11	121.6 (2)
C3—C4—C5	127.5 (2)	C16—C12—C11	121.0 (2)
C3—C4—H4	116.3	C12—C13—C14	119.3 (2)
C5—C4—H4	116.3	C12—C13—H13	120.3
C10—C5—C6	117.9 (2)	C14—C13—H13	120.3
C10—C5—C4	119.1 (2)	N3—C14—C13	123.6 (2)
C6—C5—C4	123.0 (2)	N3—C14—H14	118.2
C7—C6—C5	121.1 (2)	C13—C14—H14	118.2
C7—C6—H6	119.4	N3—C15—C16	123.4 (2)
C5—C6—H6	119.4	N3—C15—H15	118.3
C6—C7—C8	120.2 (2)	C16—C15—H15	118.3
C6—C7—H7	119.9	C12—C16—C15	119.4 (2)
C8—C7—H7	119.9	C12—C16—H16	120.3
C7—C8—C9	119.7 (2)	C15—C16—H16	120.3
C7—C8—H8	120.2		
			
C1—N1—N2—C2	−176.15 (19)	C8—C9—C10—C5	−0.5 (4)
N2—N1—C1—S2	−177.22 (15)	C6—C5—C10—C9	−0.5 (3)
N2—N1—C1—S1	2.5 (2)	C4—C5—C10—C9	179.6 (2)
C11—S1—C1—N1	175.41 (16)	C1—S1—C11—C12	−174.77 (15)
C11—S1—C1—S2	−4.84 (16)	S1—C11—C12—C13	−103.5 (2)
N1—N2—C2—C3	−177.19 (18)	S1—C11—C12—C16	73.2 (2)
N2—C2—C3—C4	179.2 (2)	C16—C12—C13—C14	0.1 (4)
C2—C3—C4—C5	−176.9 (2)	C11—C12—C13—C14	176.9 (2)
C3—C4—C5—C10	179.6 (2)	C15—N3—C14—C13	−0.1 (4)
C3—C4—C5—C6	−0.2 (4)	C12—C13—C14—N3	0.0 (4)
C10—C5—C6—C7	1.3 (4)	C14—N3—C15—C16	0.2 (4)
C4—C5—C6—C7	−178.8 (2)	C13—C12—C16—C15	0.0 (3)
C5—C6—C7—C8	−1.1 (4)	C11—C12—C16—C15	−176.9 (2)
C6—C7—C8—C9	0.1 (4)	N3—C15—C16—C12	−0.1 (4)
C7—C8—C9—C10	0.8 (4)		

### Hydrogen-bond geometry (Å, º)

Cg1 is the centroid of the N3,C12–C15 pyridyl ring.

**Table:**

*D*—H···*A*	*D*—H	H···*A*	*D*···*A*	*D*—H···*A*
N1—H1*n*···N3^i^	0.88 (2)	2.02 (2)	2.897 (3)	172 (2)
C8—H8···*Cg*1^ii^	0.95	2.92	3.701 (3)	141

Symmetry codes: (i) *x*+1, *y*−1, *z*; (ii) −*x*+2, −*y*+1, −*z*+1.
